# Antibacterial activity and mode of action of acetone crude leaf extracts of under-investigated *Syzygium* and *Eugenia* (Myrtaceae) species on multidrug resistant porcine diarrhoeagenic *Escherichia coli*

**DOI:** 10.1186/s12917-019-1914-9

**Published:** 2019-05-22

**Authors:** Ibukun M. Famuyide, Abimbola O. Aro, Folorunso O. Fasina, Jacobus N. Eloff, Lyndy J. McGaw

**Affiliations:** 1 Phytomedicine Programme, Department of Paraclinical Sciences, Faculty of Veterinary Science, University of Pretoria, Private Bag X04, Onderstepoort, Pretoria, 0110 South Africa; 2 Department of Veterinary Tropical Diseases, Faculty of Veterinary Science, University of Pretoria, Private Bag X04, Onderstepoort, Pretoria, 0110 South Africa; 3 Emergency Center for Transboundary Animal Diseases-Food and Agriculture Organization of the United Nations, Dar es Salaam, Tanzania

**Keywords:** Antimicrobial activity, Diarrhoea, Enterotoxigenic *E. coli*, Anti-adhesion, Phytogenic feed alternative, Plant extracts, *Eugenia*, *Syzygium*, Cytotoxicity

## Abstract

**Background:**

Diarrhoea, a global economically important disease burden affecting swine and, especially piglets, is commonly caused by infection with entero-toxigenic *E. coli* (ETEC). Adherence of ETEC to porcine intestinal epithelial cells following infection, is necessary for its pathogenesis. While antimicrobials are commonly given as therapy or as feed additives for prophylaxis against microbial infections, the concern over increased levels of antimicrobial resistance necessitate the search for safe and effective alternatives in livestock feed. Attention is shifting to natural products including plants as suitable alternatives to antimicrobials.

The activity of acetone crude leaf extracts of nine under-explored South African endemic plants from the Myrtaceae family with good antimicrobial activity were tested against pathogenic *E. coli* of porcine origin using a microplate serial dilution method. Bioautography, also with p-iodonitrotetrazolium violet as growth indicator was used to view the number of bioactive compounds in each extract. In vitro toxicity of extracts was determined against Caco-2 cells using the 3-(4,5-dimethythiazolyl-2)-2,5-diphenyltetrazolium bromide reduction assay. The antimicrobial susceptibility of *E. coli* isolates was tested on a panel of antimicrobials using the Kirby-Bauer agar diffusion method while the anti-adherence mechanism was evaluated using a Caco-2 cell enterocyte anti-adhesion model.

**Results:**

The MIC of the extracts ranged from 0.07–0.14 mg/mL with *S. legatii* having the best mean MIC (0.05 mg/mL). Bioautography revealed at least two active bands in each plant extract. The 50% lethal concentration (LC_50_) values ranged between 0.03–0.66 mg/mL. *Eugenia zeyheri* least cytotoxic (LC_50_ = 0.66 mg/ml) while *E. natalitia* had the highest cytotoxicity (LC_50_ = 0.03 mg/mL). All the bacteria were completely resistant to doxycycline and colistin sulphate and many of the plant extracts significantly reduced adhesion of *E. coli* to Caco-2 cells.

**Conclusions:**

The extracts of the plants had good antibacterial activity as well as a protective role on intestinal epithelial cells against enterotoxigenic *E. coli* bacterial adhesion. This supports the potential use of these species in limiting infection causes by *E. coli*. Some of these plants or extracts may be useful as phytogenic feed additives but it has to be investigated by animal feed trials.

## Background

Diarrhoea poses a significant limitation to the progress being made in the swine production industry globally. It causes huge economic losses, reduces growth rate and causes high treatment costs [1]. The mean direct economic cost per annum in China alone due to swine diarrhoea is approximately US$ 145 million [2]. Other countries such as the USA and Netherlands, with huge swine industries also experience a high percentage of piglet mortality annually due to diarrhoea [2]. In South Africa, the pork industry accounts for about 2.15% of the domestic agricultural sector [3], and makes up about 0.2% of global pork production [4]. Diarrhoea is identified as a major disease that limits efficient pork production in South Africa [5].

Colibacillosis is a diarrhoeic disease caused by pathogenic strains of *E. coli*, a Gram-negative bacterial species [6]. Enterotoxigenic *E. coli* (ETEC) and shiga toxin producing *E. coli* (STEC) mostly account for neonatal and post-weaning diarrhoea in piglets [4, 7]. An array of virulence genes carried by ETEC are responsible for their pathogenicity. These are classified as fimbrial (F4, F5, F6, F17, F18, F41), toxin (LT, ST, STx2e, EAST 1) and adhesin (AIDA-1, paa) genes [8, 9]. Through the adhesin molecules, the bacteria attach to, and colonize the intestinal cells to establish infection and produce toxins that eventually cause the diarrhoea symptoms [10, 11]. Adherence of bacteria to cells is necessary for cellular invasion [12].

Antimicrobials are widely used against bacterial infections in animals either for therapeutic treatment, or for prophylaxis by sub-therapeutic inclusions in animal feeds [13]. Antibiotic-containing feed is beneficial to animal health by promoting growth and reducing the risk of occurrence and severity of gastrointestinal disorders, especially those caused by pathogens such as *E. coli* [14]. Their use has however contributed to the development and increase in levels of antimicrobial resistance (AMR) in both veterinary and human medicine [15, 16]. Important pathogens like *Campylobacter* spp., *Salmonella* spp., *Escherichia coli* and *Enterococcus* spp., have poor responses to antibiotic therapy [17]. Previous reports show that ETEC from humans and animals have multiple resistance to a wide range of antimicrobials. In one study, tetracycline, ampicillin, and trimethoprim-sulphamethoxazole had population resistance values of 66.7, 61.1, and 58.3% respectively [18]. Other commonly used antimicrobials in animal feed include colistin sulphate, avilamycin, monensin and salinomycin. However, most of these agents have been banned for use as feed additives, especially in the European Union. China has banned the use of colistin as feed additive in its livestock industry [19]. Colistin, an antimicrobial which is a last-line treatment option for multidrug-resistant Gram-negative bacterial infections in humans [20] is commonly used as antibiotic additive in livestock feeds [13]. Members of the Enterobacteriaceae from humans and animals carry the MCR-1 gene which confers resistance against colistin [21, 22].

In view of the risks associated with antimicrobial inclusion in animal feeds, there is enough motivation to focus on the search for alternative, non-antibiotic, but equally effective products that can serve as antibiotic feed replacers in animal production, especially in swine [23]. Several feed additives have been reported to be potential antibiotic replacers in animal feed. Examples include enzymes, organic acids, prebiotics, probiotics, organic minerals, oligosaccharides, toxin binders, and phytogenic feed additives [17].

Some plant metabolites known as phytogenics represent potential novel therapeutic options available for study and development as effective feed additives. Many plant extracts, and plant-derived products such as essential oils hold promise to enhance the growth and health of production animals when added to their feed, and these are comparable in efficacy to conventional feed antimicrobials [24]. The interest in plants is due to a wide array of biological activities that may improve animal health. Such activities include suppression of growth of pathogenic microbes due to their wide range of antimicrobial activities against Gram- positive and Gram-negative bacteria, fungi, viruses and other pathogenic parasites affecting livestock [25, 26]. Plant crude extracts and isolated compounds may also have immune modulating activity due to their strong effect on oxidation and inflammation [27]. These observed biological effects of plants from in vitro and in vivo observations provides evidence to support their inclusion in livestock feed, especially as replacements for antimicrobials and as growth promoters. For example, in one study [28], the addition of seed extracts of black cumin, fenugreek and black tea leaf extracts enhanced the production performance and microbial health of laying hens. In another study, the inclusion of cinnamaldehyde and other additives in pig feeds decreased *Salmonella* Typhimurium faecal shedding and improved pig weight gain [29]. Furthermore, licorice and *Macleaya cordata* (Papaveraceae), which are phytogenic feed additives approved for livestock feeding in the European Union [30, 31] show that plant extracts have potential to replace antimicrobials in animal feed. Apart from their influence on animal performance, plant additives may also have a positive influence on environmental health by reducing noxious gas content in animal faeces and rumen gases thereby reducing the concentration and effects of greenhouse gases [32]. Sows fed with leaf material of *Origanum vulgare* showed improved growth and reproductive performance [33]. In South Africa, many small-scale livestock farmers, especially the ones in rural communities with little access to veterinary services, depend on plants and other traditional remedies for the health management of their animals [34]. For example, livestock farmers in the Eastern Cape region of South Africa use plants to treat sickness such as diarrhoea, intestinal worms, inflammation, gall sickness and Redwater sickness [35].

Little information is available on the scientific investigation of the antimicrobial activity of indigenous South African medicinal plants on pathogens of veterinary importance, especially those attempting to understand the mode of action focused on the ability of such plant extracts or compounds to reduce adhesion or invasion of diarrhoeagenic bacteria such as *E. coli* to intestinal cells. Caco-2 cells (a human adenocarcinoma cell line) are a commonly used model to study interactions in the gut because of their resemblance to enterocytes that line the intestine of monogastric animals [36]. Studies have demonstrated the ability of *E. coli* to adhere to and invade human colonic adenocarcinoma cells such as Caco-2 cells [37–39]. Previous research in our group revealed that plant species from the family Myrtaceae had promising activity against *E. coli* [40]. Based on this, we selected nine plant species from the *Syzygium* and *Eugenia* genera in the Myrtaceae for this study.

The Myrtaceae family consists of shrubs and trees with approximately 145 genera and over 5500 species found in the tropics and sub-tropical regions all over the world [41]. The *Eugenia* genus comprises approximately 600 species of which at least 14 are present in southern Africa [42] while the *Syzygium* genus consists of about 500 species of evergreen flowering plants and the fruits of many of the plants are edible [43]. The traditional uses, phytochemistry, antimicrobial activities and toxicological activities of many plants in these genera have been reported while extensive studies abound on famous ones such as *S. aromaticum* (clove) and *E. uniflora* (pitanga) [44–46]. In South Africa, few pharmacological studies exist on plants from the *Syzygium* and *Eugenia* genera of the Myrtaceae family. One of the South African native plants, *Syzygium cordatum* has been used traditionally against diarrhoea and tuberculosis with reported pharmacological activities [47, 48]. The dearth of information presents an opportunity to study other relatively unknown and understudied South African plants from both genera for their pharmacological activities. The aim of this study is to determine the antibacterial activity, and safety of acetone crude leaf extracts of nine under-studied plants from the *Eugenia* and *Syzygium* genera (Myrtaceae) against some resistant enterotoxigenic *E. coli* strains and to determine whether the extracts could interfere with growth or inhibit *E. coli* adherence to intestinal cells, thereby reducing *E. coli* infections in food animals and subsequent transmission to humans. The human colon adenocarcinoma cell line (Caco-2) was used as an enterocyte model for the adherence assays.

## Results

### Extract yield and total activity

The extraction of the nine plants with acetone gave different yields (Table 1). The highest yield was obtained from *Eugenia zeyheri* (25.33%) followed by *E. erythrophylla* (18.50%).

**Table 1:** Percentage yield, minimum inhibitory concentration (MIC), and total antibacterial activity (TAA) of nine acetone leaf extracts against six *E. coli* clinical strains and a reference strain (ATCC 25922). Bold numbers show good MIC values

	ETEC strains with virulence genes													*E. coli* (ATCC)	
		STA, F6		EAST1		STA		STX2E, STA		Sta, EAST-1, AIDA 1		STA, STB, LTB, EAST 1, O149, F4			
Plant	% yield	MIC (mg/mL	TAA (mL/g)	MIC (mg/mL)	TAA (mL/g)	MIC (mg/mL)	TAA (mL/g)	MIC (mg/mL)	TAA (mL/g)	MIC (mg/mL)	TAA (mL/g)	MIC (mg/mL)	TAA (mL/g)	MIC (mg/mL)	TAA mL/g)
*E. erythrophylla*	18.5	0.16	1184	**0.04**	4736	**0.08**	2368	**0.08**	2368	**0.08**	2368	0.23	789	0.10	1776
*E. natalitia*	9.4	**0.08**	1203	**0.08**	1203	**0.04**	2406	**0.08**	1203	**0.08**	1203	0.16	601	0.16	602
*E. woodii*	9.8	**0.08**	1254	**0.08**	1254	**0.08**	1254	**0.08**	1254	**0.08**	1254	**0.08**	1254	0.16	627
*E. umtamvunensis*	8.77	0.16	561	**0.08**	1122	**0.08**	1122	0.16	561	**0.04**	2244	0.23	374	0.31	281
*E. zeyheri*	25.33	**0.04**	6485	**0.08**	3243	**0.08**	3243	0.16	1621	0.16	1621	0.23	1081	0.13	1946
*S. legatii*	9.23	**0.04**	2364	**0.04**	2364	**0.04**	2364	**0.04**	2364	**0.04**	2364	**0.06**	1576	**0.08**	1182
*S. masukuense*	10.5	**0.08**	1344	**0.04**	2688	**0.04**	2688	0.16	672	**0.08**	1344	**0.08**	1344.00	**0.08**	134
*S.* species A	10.65	**0.08**	1363	**0.04**	2726	**0.04**	2726	**0.04**	2726	**0.04**	2726	0.12	908.80	**0.08**	1363
*S. gerrardii*	17	0.16	1088	0.31	544	0.16	1088	**0.04**	4352	0.31	544	**0.06**	2901.33	0.31	544
Tetracycline	NA	0.31	NA	1.25	NA	0.31	NA	1.25	NA	0.31	NA	0.31	NA	**0.02**	NA
Average for extracts	NA	**0.10**	NA	**0.09**	NA	**0.07**	NA	**0.09**	NA	**0.10**	NA	0.14	NA	0.16	NA

### TLC and bioautography

**Fig. 1:**
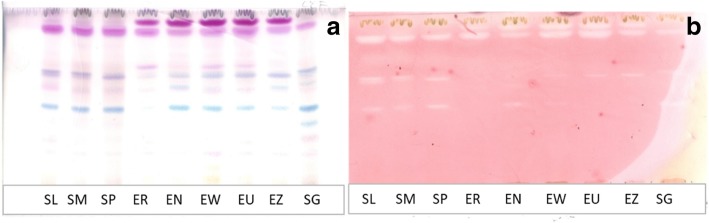
**a** Chromatogram developed in chloroform: ethyl acetate: formic acid (5:4:1, CEF) solvent system of the acetone leaf extracts of the nine plants sprayed with vanillin. **b** Bioautograms of *Escherichia coli* inhibition developed with CEF; white bands indicate compounds that inhibit the growth of the bacteria. SL = *Syzygium legatii*, SM = *Syzygium masukuense*, SP = *Syzygium* sp., SG = *Syzygium gerrardii*, ER = *Eugenia erythrophylla*, EN = *Eugenia natalitia*, EW = *Eugenia woodii*, EU = *Eugenia umtamvunensis,* EZ = *Eugenia zeyheri*

Phytochemical screening of the plant acetone extracts in this study using thin layer chromatography showed different chemical components as depicted by the different colours (Fig. 1a). The active compounds against *E. coli* were best separated by the intermediate solvent system, chloroform: ethylacetate: formic acid (CEF) (Fig. 1a). Most of the active compounds may be of intermediate polarity. The parts of the chromatogram showing white clear zones surrounded by pinkish areas shows the R_f_ values of active compounds (Fig. 1b). More than one active compound was observed for most of the extracts. The retention factor (R_f_) of the active compounds was obtained by dividing the distance moved by the compound by the solvent distance. One active band (R_f_ value = 0.88) was observed in all the plant crude extracts. *S. masukuense* and *S. legatii* extracts had the highest number of active bands (4) from this study with R_f_ values of 0.88, 0.73, 0.72 and 0.53. The *S*. sp. extract had all these compounds except for one (R_f_ = 0.73). *Syzygium gerrardii*, *E. umtamvunensis* and *E. zeyheri* had similar active bands (R_f_ = 0.94, 0.88, 0.71). Also, *E. erythrophylla* and *E. woodii* had two active compounds (R_f_ = 0.94 and 0.88). *Eugenia natalitia* extracts had three active bands (R_f =_ 0.94, 0.88, 0.53) while two active compounds (R_f_ = 0.94, 0.88) were present in all the species from the *Eugenia* genus. Also, compounds with R_f_ values of 0.88 and 0.71 were present in all plants in the *Syzygium* genera.

### Antimicrobial susceptibility

**Fig. 2:**
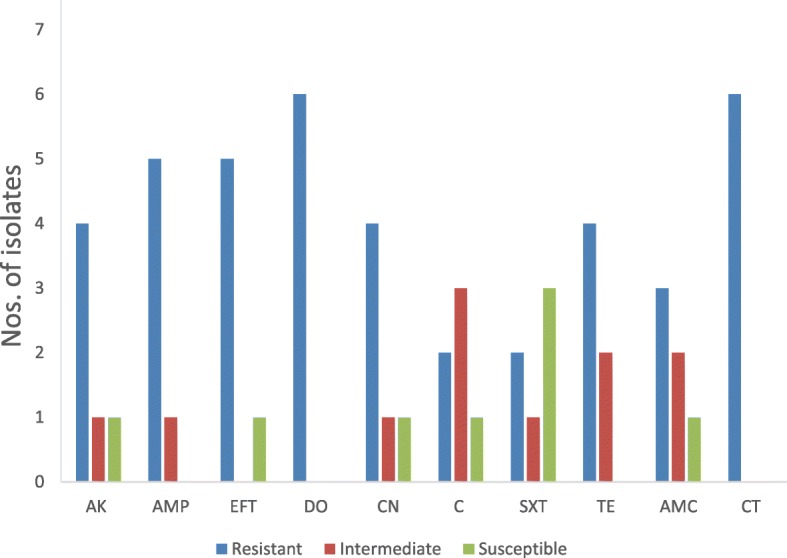
Antimicrobial susceptibility of ETEC isolates to 10 widely-used antibiotics. AK = Amikacin, AMP = Ampicillin, EFT = Ceftiofur, DO = Doxycycline, CN = Gentamicin, C = Chloramphenicol, SXT = Sulphamethoxazole/trimethoprim, TE = Tetracycline, AMC = Amoxicillin/clavulanic acid, CT = Colistin sulphate

The susceptibility of the six *E. coli* clinical strains used in this study against selected antimicrobials is presented in Fig. 2. A panel of ten antimicrobials belonging to different antibiotic classes was tested. Amikacin and gentamicin are aminoglycosides, while doxycycline and tetracycline are grouped as tetracycline class of antimicrobials. Ampicillin as well as amoxycillin/clavulanic acid are penicillin while ceftiofur is a cephalosporin. Also, colistin sulphate is a polymyxin antibiotic, while chloramphenicol and sulphamethoxazole/trimethoprim are classified as polymyxin and β-lactamase inhibitors respectively [49]. These antimicrobials or their modified/combination mix are commonly used in animal production either for prophylaxis or for therapy in clinical conditions. All the bacteria were completely resistant to doxycycline and colistin sulphate. Five isolates were resistant to ceftiofur and ampicillin, while four of the isolates were resistant to amikacin, tetracycline and gentamicin respectively. Three isolates were resistant to amoxycillin/clavulanic acid while two isolates showed resistance to sulphamethoxazole/trimethoprim and chloramphenicol respectively. Three isolates were susceptible to sulphamethoxazole/trimethoprim. One of the isolates, having the toxin gene STA, was completely resistant to all the antibiotics.

### Antibacterial activity (minimum inhibitory concentration) and total antibacterial activity

**Fig. 3:**
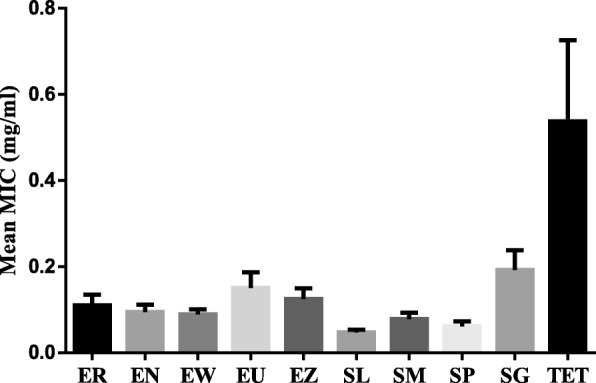
The mean MIC in mg/mL of the acetone leaf extracts of the nine plants against seven *E. coli* strains. ER = *Eugenia erythrophylla*, EN = *Eugenia natalitia*, EW = *Eugenia woodii*, EU = Eugenia umtamvunensis, EZ = *Eugenia zeyheri*, SL = *Syzygium legatii*, SM = *Syzygium masukuense*, SG = *Syzygium gerrardii*, TET = Tetracycline

The antibacterial activity of the nine selected plants extracts against the different strains of *E. coli* is shown in Table 1. The MIC values for the extracts ranged between 0.04–0.31 mg/mL. The values for tetracycline ranged from 0.02–0.31 mg/mL. The clinical strains with a mean MIC range of 0.07–0.14 mg/mL were more sensitive to the extracts than the reference *E. coli* (MIC = 0.16 mg/mL). Of the nine plants, *S. legatii* extract had the best mean MIC (0.05 mg/mL) against all *E. coli* strains investigated in this study followed by *S. masukuense* (MIC = 0.06 mg/mL) and *S*. sp. (MIC = 0.08 mg/mL) extract. *Eugenia natalitia* and *E. woodii* extracts had a mean MIC of 0.09 mg/mL against the bacteria strains, while *E. erythrophylla, E. umtamvunensis*, *E. zeyheri* and *S. gerrardii* extracts had mean MICs of 0.11 mg/mL, 0.15 mg/mL, 0.12 mg/mL, and 0.19 mg/mL respectively. The antibiotic, tetracycline had very low activity with a mean MIC of 0.54 mg/mL against the isolates (Fig. 3). In this study, *E. zeyheri* had the highest mean total antibacterial activity of 2.75 L/g. This means that the acetone extract of 1 g of acetone crude extract of *E. zeyheri* can be diluted to 2.75 L and still retain the ability to inhibit bacterial growth. *Eugenia umtamvunensis* had the lowest mean total antibacterial activity of 0.9 L/g.

### Cytotoxicity

The cytotoxicity results against Caco-2 cells are presented in Table 2. The LC_50_ values ranged between 0.03–0.6 mg/mL. *E. natalitia* had the highest toxicity (LC_50_ = 0.03 mg/mL) while *E. zeyheri* had the lowest toxicity i.e. highest LC_50_ value of 0.66 mg/mL. The cytotoxicity (mg/mL) and MIC (mg/mL) values are both used to calculate the selectivity index (SI) of a plant extract, which is a measure of the safety of the extract [50]. The results for the SI are shown in Table 2. *E. zeyheri* had the best selectivity index of 7.17 against all the pathogens followed by *E. umtamvunensis* (6.01), *E. erythrophylla* (3.53), *S. legatii* (3.94) and *S. gerrardii* (3.69). *E. natalitia* had the lowest selectivity index of 0.45.

**Table 2:** Cytotoxicity against Caco-2 cells (LC_50_, mg/mL) and Selectivity Index (SI) of the nine selected acetone crude extracts. Selectivity index values ≥1 show that an extract is more toxic to the pathogens than to the mammalian cells. The higher the SI, the less cytotoxic the extract, indicating possible enhanced safety

		Selectivity index (SI)							
	Cytotoxicity	STA, F6	EAST1	STA	STX2E, STA	Sta, EAST-1, AIDA 1	STA, STB, LTB, EAST 1, O149, F4	*E. coli* (ATCC)	Mean SI
*E. erythrophylla*	0.29	1.84	7.35	3.68	3.68	3.68	1.23	2.76	3.46
*E. natalitia*	0.03	0.45	0.45	0.89	0.45	0.45	0.22	0.22	0.45
*E. woodii*	0.18	2.25	2.25	2.25	2.25	2.25	2.25	1.12	2.09
*E. umtamvunensis*	0.59	3.76	7.53	7.53	3.76	15.06	2.51	1.88	6.01
*E. zeyheri*	0.66	16.91	8.46	8.46	4.23	4.23	2.82	5.07	7.17
*S. legatii*	0.17	4.47	4.47	4.47	4.47	4.47	2.98	2.23	3.94
*S. masukuense*	0.07	0.95	1.90	1.90	0.47	0.95	0.95	0.95	1.15
*S.* sp.	0.07	0.84	1.68	1.68	1.68	1.68	0.56	0.84	1.28
*S. gerrardii*	0.40	2.54	1.27	2.54	10.17	1.27	6.78	1.27	3.69
Doxorubicin (μM)	0.76	NA	NA	NA	NA	NA	NA	NA	NA

### Anti-adhesion activity of the plant extracts

#### Quantitative anti-adhesion evaluation

**Fig. 4:**
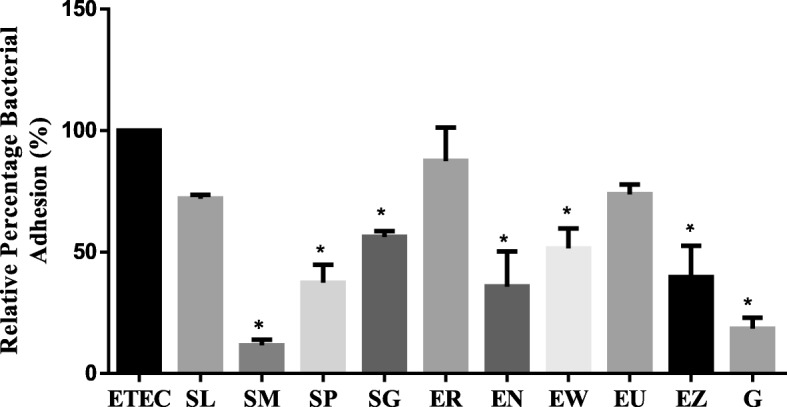
Change in *E. coli* adhesion to Caco-2 cell monolayer after treatment with acetone crude extracts of nine plants, presented as relative percentage of bacterial adhesion to cells incubated with extracts for 1 h compared with the untreated control. EE = *E. erythrophylla*; EN = *E. natalitia*; EW = *E. woodii*; EU = *E. umtamvunensis*; EZ = *E. zeyheri*; SL = S*. legatii*; SM = *S. masukuense*; SP = *S*. sp.; positive control, G = Gentamicin; untreated culture control, ETEC = Enterotoxigenic *E. coli* (virulence genes = STA, F6). Data is expressed as the mean percentage ± SEM of three replicates. Asterisks (*) above a bar indicate significant difference at *p* < 0.05, compared with the control (ETEC)

There was a difference in bacterial adhesion to Caco-2 cells incubated with crude acetone extracts of 9 plant species compared with the untreated control (Fig. 4). The relative percentage adhesion of bacteria ranged from 12 to 88%. *Syzygium masukuense* with relative percentage adhesion of 12% (i.e. 88% of cells did not adhere to Caco2 cells) was the most active plant extract. *E. erythrophylla* extract was the least active as 88% of the bacteria were still attached to Caco-2 cells. After incubation. With the exception of *S. legatii, E. erythrophylla, and E. umtamvunensis*, extracts all other extracts and gentamicin significantly (*p* < 0.05) reduced the number of bacteria that adhered to Caco-2 cells.

#### Qualitative anti-adhesion evaluation

**Fig. 5:**
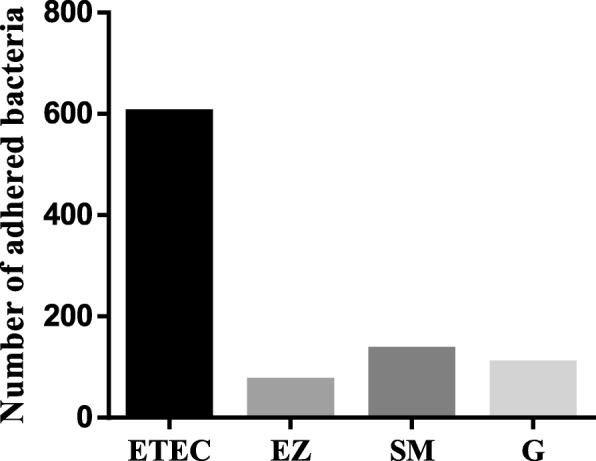
The number of adhered bacteria to Caco-2 treated cells treated with acetone crude leaf extract of two selected plants: EZ = *Eugenia zeyheri*, SL = *Syzygium masukuense*, and positive control, G = Gentamicin. ETEC (*E. coli*) = untreated control

**Fig. 6:**
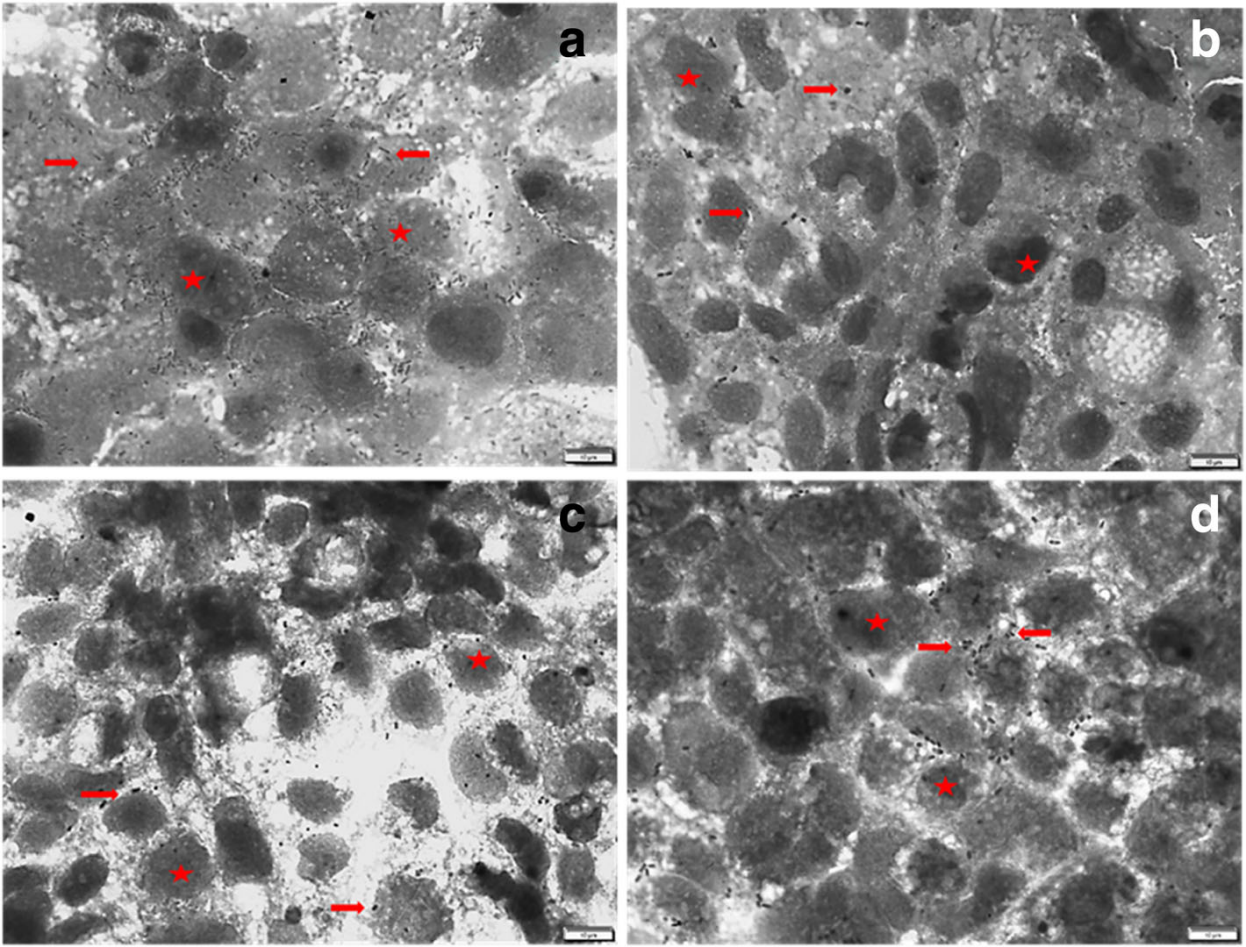
Micrograph showing the adhesion of ETEC to Caco-2 cell line in; (top left) untreated control (ETEC only), (top right) *Eugenia zeyheri* treated Caco-2 cells, (bottom left) *Syzygium masukuense* treated Caco-2 cells, (bottom right) Gentamicin treated Caco-2 cells. Cells and bacteria were stained with Giemsa and observed at 1000 × oil magnification. Arrows show adhered bacteria. Stars show Caco-2 cells

The result of the number of adhering bacteria on 20 randomly selected Caco-2 cells viewed under a light microscope is presented in Fig. 5. Two plant extracts (*S. masukuense* and *E. zeyheri*), one from each genus, were selected. Findings showed that the bacteria adhered to untreated and treated Caco-2 cells but at different degrees. Microscopically, there was an 8, 5, and 6-fold decrease in the number of attached bacteria compared to the untreated control. Figure 6 shows the microscopic appearance of adhered bacteria to Caco-2 cell treated extracts, and controls. Few bacteria were seen adhering to the Caco-2 cells compared to the untreated control.

## Discussion

This study reports on the antibacterial activity of acetone crude leaf extracts of 9 selected different plant species from the *Syzygium* and *Eugenia* (Myrtaceae) genera on multidrug resistant *E. coli* isolated from pigs. The safety and possible mode of antibacterial action were also investigated. Leaves were used in this study based on sustainable utilization concerns. Acetone was used to extract the dried leaves because acetone has been shown to be the best extractant of plant material due to the fact that it can extract compounds with a wide range of polarities, it has low toxicity to bioassay systems and it is easy to remove from plant extracts [51].

### Qualitative antibacterial assay by TLC bioautography

The bioautography results showed the different active compounds against *E. coli*. Most of the compounds in the plant extracts are of intermediate polarity. Although, it is often reported that compounds responsible for biological activities are mainly non-polar [52, 53], our results showed that active compounds can also be of intermediate polarity. The presence of similar compounds in these plants supports the fact that closely related plants may elaborate similar bioactive compounds. Related plant species may have similar composition and activity and that motivates using taxonomic parameters in selecting species for study [40]. It is possible that other active compounds may be present in the chromatograms but were obscured or not visible due to reasons such as photo-oxidation, evaporation or presence of a low amount of the active compounds [52]. It is important to note that bioautography only indicates the number of active compounds separated by TLC in an extract but it is not a quantitative measure of activity [54].

### Antimicrobial susceptibility testing

The rise in multidrug resistance in human and veterinary pathogens is on the increase globally [55] and resistant microbes could be transferred to humans by consuming animal products contaminated with antibiotic residues through the use of antibiotics as growth promoters in livestock production [56]. The *E. coli* isolates used in this study were resistant to at least 5 different antimicrobial agents, and to at least 2 different classes of antimicrobials out of the 7 classes used in this study. Most (83.3%) of the isolates were multidrug resistant. The strains were completely resistant to doxycycline and colistin sulphate which is extremely concerning. Tetracycline is a commonly used antimicrobials in animal production, and has been detected in different levels in animal feed, and tissues [57]. Colistin sulphate, a polymyxin antibiotic is commonly used as a feed additive and in chemotherapy to treat gastro-intestinal diseases in livestock caused by pathogens such as *E. coli* [22]. The resistance to colistin sulphate shown by all the isolates in this study is worrisome as this antibiotic is a last resort to treat human multidrug resistant pathogens [58]. In a recent study, resistance to colistin sulphate by *E. coli* isolated from pigs was high [59]. The resistance displayed by the *E. coli* isolates in this study is likely to be connected with the presence of resistance genes such as the MCR-1 gene which has been reported in resistant *E. coli* [59]. Hence, a strong case has been made to totally stop the use of antibiotics in animal feeds [60], while many alternatives including phytogenics/botanicals have been proposed [61].

### Minimum inhibitory activity and total antibacterial activity of plant extracts

In this study, the minimum inhibitory concentration of the selected plant extracts was determined using the widely used broth microdilution method where the lower the MIC value of a plant extract is, the better is the activity [62]. The MIC of an extract is regarded as good if the values are less than 0.1 mg/mL, moderate if it is between 0.1 and 0.625 mg/mL, and weak when it is above 0.625 mg/mL [63]. Based on this, the MIC of the acetone crude extract of the nine plants against the *E. coli* strains ranged from good to moderate. *S. legatii* had the best mean MIC of 0.05 mg/mL against the *E. coli* strains (Fig. 3), compared to the MIC of other extracts, and Tetracycline (0.19 mg/mL). This study reports for the first time, the antibacterial activity of the acetone crude leaf extracts of the studied nine plant sp. against diarrhoeagenic *E. coli*. Scanty studies exist on the selected plants with which we can compare our findings. For example, a study [64] reported that the essential oil of *E. natalitia* had an MIC value of 0.1 mg/mL against *E. coli*.

Other reports on antibacterial activity of other species in the *Syzygium* and *Eugenia* genera of the Myrtaceae family are available and results are comparable to our study as MIC values are reported. For example, dichloromethane: methanol crude bark extracts of *Syzygium cordatum* had an MIC of 1.20 mg/mL against *E. coli* [48]. Another study reported MIC values of 0.156 mg/mL and 0.321 mg/mL for ethanol and methanol crude leaf extracts for *S. cordatum* [65]. In another study on Australian *Syzygium* species, the methanol fruit extracts of *S. australe* had a MIC of 2.5 mg/mL against *E. coli* while the ethyl acetate extract of *S. leuhmannii* had an inhibitory concentration of 0.8 mg/mL against *E. coli* [66]*.* The hydro-alcoholic crude extract of *Eugenia brasiliensis* had an MIC of > 2 mg/mL against *E. coli* (ATCC 43895) [67].

Compared to tetracycline, the crude acetone extracts of all the plants in this study had significantly superior antibacterial activity (*p* < 0.05). This observation is supported by the antimicrobial susceptibility profile where most of the *E. coli* strains were resistant to tetracycline. Resistance to tetracycline in livestock, especially in pigs, is widely reported in literature [68].

The potency of a plant extract may be predicted on the basis of its MIC in mg/mL. The best plant species to be used is based on the total antibacterial activity (TAA) [62]. It is calculated by dividing the mg extracted from one g of plant material with the MIC. The TAA gives the volume (mL) to which the extract obtained from 1 g of plant material can be diluted and still be able to inhibit the bacterial growth. The TAA is also useful to determine the most suitable extract for compound isolation and bioprospecting [69]. Based on this study, *E. zeyheri* had the highest TAA of 2.75 L/g.

### Cytotoxicity of plant extracts

Although it is often assumed that plant extracts and other natural products are safe, it is necessary to determine their cytotoxicity to provide preliminary scientific evidence whether they are safe or not [50]. Additionally, the biological activity seen in a plant extract may be as a result of a toxic principle in the plant [53]. A plant extract is considered to be cytotoxic when the LC_50_ is 20 μg/mL and below [70]. All of the plant extracts in this study had LC_50_ values greater than 20 μg/mL on Caco-2 cells. It should however be borne in mind that in vitro cellular toxicity may not equate to whole animal toxicity because of different factors such as gut interactions and bioavailability. Therefore, acute and chronic animal toxicity studies are needed to determine the toxicity of the extracts [71]. A SI value greater than 1 is considered the benchmark for the usefulness of an extract. It means that the extract is more toxic to the pathogen than to normal body cells. The higher the SI value, the safer is the plant extract and the higher is the possibility of developing a herbal product from the plants [69]. *Eugenia zeyheri* was the least cytotoxic while *E. natalitia* was the most toxic (LC_50_ = 0.03 mg/mL) to Caco-2 cells. In a study, essential oil from leaves of *E. natalitia* was reported to be cytotoxic with LC_50_ value of 0.018 mg/mL using the brine shrimp mortality assay [64]. With most (88.9%) of the plants investigated having SI values > 1 (Table 2), the potential of developing them as a phytogenic feed additive is reasonable. Otherwise, it is also possible to isolate active compounds which can form a template for the development of new antibacterial drugs.

### Anti-adhesion assays

Few studies are available on the mode of action of bioactive plants and to our knowledge, no study has been carried out to evaluate the anti-adherence activity of the plant extracts used in this study. We to investigate if extracts interfere with the initial process of bacterial adhesion. This is an important initial strategy for diarrhoeagenic bacteria in their pathogenesis [72], and may be a mechanism of action of the plant extracts. Targeting bacterial adhesion may be a novel tool for drug discovery and development to deal with the selection pressure for resistance that often occurs when there is microbicidal action against pathogenic microbes [72]. Six of the nine tested plant extracts significantly reduced E. coli adhesion to Caco-2 cells. Of these, *S. masukuense* had the best anti-adhesion activity.

The microscopy images showed that the *E. coli* was able to adhere to Caco-2 cells (Fig. 6). The ability to adhere is most likely due to the presence of the adhesin gene present in the bacteria. Adhesions, fimbrial or non-fimbrial, are mainly responsible for attachment of pathogenic *E. coli* to the intestinal cells in diarrhoea pathogenesis [7, 73]. Many of the extracts led to significant reduction in the percentage of adhered pathogenic *E. coli* to Caco-2 cells (Fig. 4). Reduced adhesion of ETEC to the enterocytes may be due to the action of phytochemicals in the extracts such as polyphenols and tannins which have been reported to bind adhesins and bacterial cells walls, inhibit enzymes, disrupt cell membranes, as well as form complexes with metal ions [74]. It is also possible that the extracts are able to block specific receptors on the cells, thereby preventing adhesion of the bacteria. The extracts may also have interfered with bacterial adhesion by binding to receptors on the bacteria itself or by downregulating the expression of binding factors in the bacteria [75]. More studies are needed to elucidate the mechanism of action.

## Conclusion

In this study, acetone crude extracts of the plant species investigated had good in vitro antibacterial activity against some multi-drug resistant *E*. *coli* cultures. The extracts may also act as anti-adhesion agents due to their good anti-adherence activity as observed on Caco-2 infected cells.

To the best our knowledge, this is the first report on the antibacterial activity and mechanism of anti-adherence against multidrug resistant clinical isolates of *E. coli* of veterinary importance by extracts of South African plants from the Myrtaceae family. Since the search for non- antibiotic alternatives to antimicrobials is still on-going, particularly to combat the issues of antimicrobial resistance, some of the plant species used in this study, especially *E. zeyheri* may be a useful natural resource to study further for development as phytogenic feed additives. It will be of interest to explore the possible morphological damage to the bacteria by the bioactive plants using electron microscopy to further elucidate their antimicrobial action. A further study may require determining the in vivo toxicity of interesting plant extracts from this study as well as performing animal feed trials on bioactive and safe plant extracts.

## Methods

### Plant collection, drying and storage

The plants were collected in March 2017 from the Lowveld National Botanical Garden in Nelspruit, Mpumalanga, South Africa after appropriate permits were received. Herbarium specimens of the plants were prepared and deposited in the HGWJ Schweickerdt Herbarium of the University of Pretoria where identification was confirmed and voucher specimen numbers (PRU) obtained. The names of the plants and assigned voucher numbers were: *Eugenia erythrophylla* Strey (PRU 123616), *Eugenia natalitia* Sond. (PRU 123613), *Eugenia woodii* Dummer (PRU 123615), *Eugenia umtamvunensis* A.E.van Wyk (PRU 123618), *Eugenia zeyher*i (Harv.) Harv. (PRU 123617), *Syzygium legati*i Burtt Davy & Greenway (PRU 123619), *Syzygium masukuense* (Baker) R.E.Fr. ssp. *masukuense* (PRU 123623), *Syzygium* sp. (a new undescribed species) (PRU 123622) and *Syzygium gerrardii* (Harv. Ex Hook.f.) Burtt Davy (PRU 123620). The plants are mainly distributed in the Eastern Cape, KwaZulu-Natal, Mpumalanga, and Limpopo provinces of South Africa [76]. After collection in loose wire mesh orange bags, the plants were processed according to protocols developed in the Phytomedicine Programme of the Faculty of Veterinary Science, University of Pretoria [40]. In brief, clean, healthy leaves were stored indoors in a well-ventilated room at room temperature to facilitate air drying and reduction of microbial attack. The dried leaves were then ground to a fine powder using a Janke and Künkel homogenizer Model A10 mill. The leaf powders were weighed and stored in closed glass containers in the dark at room temperature.

### Plant extraction

Acetone (technical grade, Merck) was used to extract the ground leaves of the plants using a ratio of 1:10 of leaf material to extractant [77]. Briefly, 2 grams (2 g) of each tree leaf sample were extracted with 20 mL acetone. The mixture was sonicated for 20 min, vigorously shaken, and then poured into a 50 mL polyester centrifuge tube and centrifuged at 4000 x g for 10 min (Hettich Centrifuge, Rotofix 32 A, Labotec, Johannesburg, South Africa). The supernatant was collected and filtered through Whatman No. 1 filter paper into pre-weighed glass vials and concentrated by drying under a stream of cold air. Then the dried extracts were weighed and the yield calculated by dividing the mass extracted by the initial mass. A concentration of 10 mg/mL (stock solution) in acetone was prepared for use in the assays.

### Analysis of extracts by thin layer chromatography (TLC)

Thin layer chromatography (TLC) fingerprints of the extracts were obtained on aluminum-backed silica gel plates as previously described [51]. Three different solvent systems of diverse polarities namely benzene: ethanol: ammonium hydroxide (90.10:1, BEA, non-polar basic); chloroform: ethylacetate: formic acid (5:4:1, CEF, intermediate polarity, acidic) and ethylacetate: methanol: water (40:5.4:5, EMW, polar, neutral) were used to analyze 100 μg (10 μL of 10 mg/mL) of the extract loaded in a band of 1 cm width on the TLC plates (Merck aluminium-backed plates, silica gel 60 F_254_). Visible bands were marked under white light and ultraviolet light (254 nm and 360 nm wavelengths, Camac universal UV light lamp TL-600) before being sprayed with freshly prepared vanillin (0.1 g vanillin, 28 mL methanol, 1 mL sulphuric acid) spray reagent. The plates were then heated to 110 °C for colour development.

### Test bacteria strains

The antibacterial activity of the plant extracts was determined against six enterotoxigenic *E. coli* (ETEC) clinical strains obtained from the Department of Veterinary Tropical Diseases, Faculty of Veterinary Science, University of Pretoria, South Africa. The bacteria were cultured from rectal swabs of 2-week-old piglets with diarrhoea raised on a commercial pig farm in the Gauteng province of South Africa. The organisms were isolated and cultured on MacConkey agar and pure colonies transferred to nutrient agar. The isolates were subjected to the indole test and other biochemical tests to verify *E. coli* identification. Detection of virulence genes (enterotoxin and fimbriae) were also done on the isolates using Polymerase Chain Reaction following standard protocols [78]. In addition, *E. coli* (ATCC 25922) was included as reference strain. Table 1 indicated the virulence genes of *E. coli* isolates evaluated in this study. Details of the gene has been documented in a previous report [79].

### Antimicrobial susceptibility testing

The antimicrobial susceptibility of six clinical isolates and reference *E. coli* was determined on Mueller Hinton agar plates by using Kirby-Bauer’s disc diffusion method according to Clinical and Laboratory Standards Institute (CLSI) guidelines [80] on a panel of ten antimicrobials important to human and animal health. The following antimicrobials were used: Amikacin (AK; 30 μg/disk), Ampicillin (AMP; 10 μg/disk), Amoxicillin/clavulanic acid (AMC; 30 μg/disk), Ceftiofur (EFT; 30 μg/disk), Chloramphenicol (C; 30 μg/disk), Colistin sulphate (CT; 30 μg/disk), Doxycycline (D0; 30 μg/disk), Gentamicin (CN; 10 μg/disk), Sulphamethoxazole/trimethoprim (SXT; 25 μg/disk) and Tetracycline (TE; 30 μg/disk) (Oxoid, UK). Briefly, a standard inoculum equivalent to 0.5 McFarland of *E. coli* overnight culture was inoculated on Mueller Hinton agar by even spreading and antibiotic discs were applied followed by incubation at 37 °C for 18–24 h. After incubation, the growth inhibition zones were recorded and the results were interpreted as sensitive, intermediate and resistant, in accordance to the CLSI guidelines.

### Antibacterial screening

#### Qualitative antibacterial assay by TLC bioautography

This method was applied to determine the number of active compounds in the extract and their R_f_ values. Thin layer chromatograms of the extracts were prepared as described above except that the plates were not sprayed with vanillin. The plates were allowed to dry overnight in a stream of cold air to remove the eluants. The plates were then each sprayed with an actively growing suspension of *E. coli* cultured for 18–24 h at 37 °C until they were wet. The plates were then incubated at 37 °C in a closed plastic humidified sterile container for 24 h to allow the bacteria to grow on the plates. After incubation, the plates were sprayed with 2 mg/mL of freshly prepared *p*-iodonitrotetrazolium violet (INT) (Sigma) in sterile hot distilled water and incubated further for 1–2 h for the development of clear zones against a purple-red background which suggests inhibition of bacterial growth by the compounds separated on the chromatograms [81].

### Quantitative antibacterial assay by minimum inhibitory activity and total antibacterial activity

A quick, sensitive serial dilution microplate method [82] was used to determine the minimum inhibitory concentration (MIC) of the crude plant extracts against the *E. coli* bacterial strains in triplicate in three independent experiments. The *E. coli* cultures were grown overnight in Mueller Hinton broth (Sigma Aldrich, SA) and adjusted to McFarland standard 1, equivalent to 3.7 × 10^8^ CFU/mL. The dried plant extracts were made up to a concentration of 10 mg/mL with acetone and 100 μL was added to the first well of a sterile 96-well microtitre plate and a 1:1 serial dilution was done with sterile distilled water. One hundred microlitres of the prepared bacterial cultures were added to each well. The bacteria were exposed to final extract concentrations of 2.5 to 0.02 mg/mLmL after the two-fold serial dilutions and adding the bacterial cultures. Tetracycline (Virbac, South Africa) and acetone served as positive and negative controls respectively while broth alone served as sterility control. The bacteria were exposed to 25% acetone in the first well with a two-fold decrease in subsequent wells. The microplates were then incubated overnight at 37 °C under aerobic conditions. After 16–18 h incubation, the presence of bacterial growth was detected by adding to each well 40 μl of 0.2 mg/mL INT and plates were incubated further at 37 °C for 2 h. Bacterial growth in the wells appeared as red colour which shows the reduction of INT to a red-coloured formazan. The MIC was determined visually as the lowest concentration that led to growth inhibition indicated by a reduction in the red colour [82]. The value of the total activity (mL/g) of the extracts is obtained by dividing the total mass in mg extracted from 1 g of plant material by the MIC value (mg/mL). The result indicates to what degree the active compound present in 1 g of plant material can be diluted and still kill the test organism [62, 83]

#### Cytotoxicity activity

The cytotoxicity of acetone extracts was determined against the human Caucasian colon adenocarcinoma (Caco-2, ATCC HTB 37) cell lines using the 3-(4,5-dimethylthiazol)-2,5-diphenyl tetrazolium bromide (MTT) assay [84] with few modifications [85]. The cells were grown in Dulbecco’s Modified Eagle’s Medium (DMEM, Highveld Biological, South Africa) supplemented with 10% foetal calf serum (Adcock-Ingram), 1% non-essential amino acids (Hyclone) and 1% penicillin-streptomycin (10,000 U/mL and 10 mg/mL streptomycin, Sigma) in a 5% CO_2_ incubator. Cells used were at the logarithmic phase and were between passages 30 to 40. Cell suspensions were prepared from 70 to 80% confluent monolayer cultures and seeded at a density of 1 × 10^5^ cells/mL (100 μL) in each well of sterile flat-bottomed 96-well microtitre cell culture plates and incubated for 24 h at 37 °C in a 5% CO_2_ incubator. After incubation, 100 μl of different crude plant extracts were added to the wells. Cells were exposed to the various concentrations (0.025 to 1 mg/mL) of plant extracts for 48 h. Doxorubicin (Pfizer) and acetone served as positive and negative controls respectively. After incubation for 48 h, the wells were washed with phosphate buffered saline (PBS, Sigma) and 200 μL of fresh medium was added to each well. Then 30 μL of MTT (5 mg/mL in PBS) was added to each well and the plates were incubated for 4 h at 37 °C. After this, the medium from the wells was aspirated and 50 μl of DMSO was added to the wells to solubilise the formed formazan crystals. The absorbance was measured on a microplate reader (BioTek Synergy) at a wavelength of 570 nm. The activity of each extract concentration was determined in quadruplicate and the assay was repeated three times. The concentration causing 50% inhibition of cell viability (LC_50_) was calculated. Selectivity index (SI) values for the extracts were calculated by dividing cytotoxicity LC_50_ values by the MIC values (LC_50_/MIC). The value obtained indicated the safety to efficacy ratio.

### Anti-adhesion assays

#### Quantitative anti-adhesion evaluation

The activity of the extracts on intestinal adhesion of *E. coli* was determined using the anti-adhesion assay as previously described [39, 86] with slight modifications. Caco-2 cells were maintained as described above. Cell suspensions were seeded at a density of 2 × 10^4^ cells in each well of 24-well microplates followed by incubation in 5% CO_2_ at 37 °C for 7 days. Two days before the assay, medium was aspirated from the wells, and cells were washed three times with PBS to remove antimicrobials and fresh DMEM without antimicrobials was added to wells. A day before the assay, an *E. coli* strain (having F6 fimbriae gene) was grown in Mueller Hinton (MH) broth with shaking (100 rpm) overnight at 37 °C. After 18 h, the culture was centrifuged at 5000 rpm for 10 min, pellets were resuspended in PBS supplemented with foetal bovine serum (1%, v/v) and the concentration of bacteria was adjusted to 10^9^ after measuring the optical density at 625 nm. Caco-2 cells in the 24-plate wells were infected with 500 mL of appropriately adjusted bacterial concentrations. The infected cells were treated in triplicate with the MIC concentration of the acetone crude leaf extracts of each plant. Gentamicin and acetone served as positive and negative controls respectively. After sixty minutes of incubation, the supernatant was discarded from each well and cells were washed with PBS five times to remove non- or loosely attached bacteria and to ensure that the bacteria counted were those that adhered to the cells. The cells were then lysed with 0.1% Triton-X (Sigma, St Louis, USA) at 37 °C for 5 min. After this period, the suspensions were serially diluted and 100 μL was aspirated and spread on MH agar plates in triplicate, and incubated for 24 h at 37^0^ C, followed by a viable colony count. Percentage of adhesion was calculated and compared with the control using the formula below [87]:

$$ \mathrm{Relative}\ \mathrm{percentage}\ \mathrm{of}\ \mathrm{adhesion}\ \left(\%\right)={\mathrm{CFU}}_{\mathrm{sample}}/{\mathrm{CFU}}_{\mathrm{control}}\times 100, $$

Where, CFU_sample_ is the number of bacteria adhered in wells containing sample extracts and CFU_control_ is the number of bacteria adhered in the control wells.

#### Qualitative anti-adhesion evaluation

The qualitative adhesion was determined using Caco-2 cells as previously described [39] with slight modifications. Cell suspensions were prepared from 70 to 80% confluent monolayer cultures and seeded at a density of 2 × 10^4^ cells on sterile round coverslips (13 mm diameter, Knittel-Glass, Germany) in 24-well microplates and incubated in 5% CO_2_ at 37 °C for 7 days. Two days before the assay, medium was aspirated from the wells, and cells were washed five times with PBS to remove antimicrobials and fresh DMEM without antimicrobials was added to wells. An *E. coli* strain (with the F6 fimbriae gene) was grown in MH broth with shaking (100 rpm) overnight at 37 °C for 24 h overnight. After 18 h, the culture was centrifuged at 5000 rpm for 10 min, pellets were re-suspended in PBS supplemented with foetal bovine serum (1%, v/v) and the concentration of bacteria was adjusted to 10^9^cells/mL after measuring the optical density at 625 nm. Wells were inoculated with 100 mL of appropriately adjusted bacterial concentrations and treated with acetone crude leaf extract of two selected plants (*S. masukuense* and *E. zeyheri*) at their respective MICs and allowed to interact with infected Caco-2 cells for 60 min. The plant species were selected based on their ability to significantly reduce adhesion to Caco-2 cells in the quantitative assay. Gentamicin was used as positive control. After this, cell suspensions were removed, and cells were washed with PBS to remove unattached bacteria. The bacteria and cells attached to the coverslips were then stained with Giemsa and examined under an Olympus BX63 light microscope (Olympus Corporation, Tokyo, Japan) at 100 x objective lens [88]. The number of adhering bacteria was counted on 20 randomly selected cells. Experiments were carried out in duplicate.

### Statistical analysis

Data were analyzed using Microsoft Excel and presented as mean ± SD. GraphPad prism (version 6) was used for statistical analysis. One-way analysis of variance (ANOVA) was used to analyze the data followed by Dunnett’s test. A value of *p* < 0.05 was set to determine statistical significance between the treatments and control.

## Abbreviations

ATCC: American Type Culture Collection
CFU: Colony forming unit
DMSO: Dimethyl sulphoxide
INT: *ρ*-iodonitrotetrazolium violet
LC_50_: Lethal concentration (50%)
MIC: Minimum inhibitory concentration
MTT: 3-(4,5-dimethylthiazol-2-yl)-2, 5-diphenyltetrazolium bromide
PBS: Phosphate buffer saline
SD: Standard deviation
SI: Selectivity index
STA: Heat stable enterotoxin gene A
TAA: Total antibacterial activity
